# Long non-coding RNA-MIAT promotes neurovascular remodeling in the eye and brain

**DOI:** 10.18632/oncotarget.10434

**Published:** 2016-07-06

**Authors:** Qin Jiang, Kun Shan, Xiao Qun-Wang, Rong-Mei Zhou, Hong Yang, Chang Liu, Yu-Jie Li, Jin Yao, Xiu-Miao Li, Yi Shen, Hong Cheng, Jun Yuan, Yang-Yang Zhang, Biao Yan

**Affiliations:** ^1^ Eye Hospital, Nanjing Medical University, Nanjing, China; ^2^ Research Center, Eye & ENT Hospital, Shanghai Medical College, Fudan University, Shanghai, China; ^3^ Department of Neurology, Jiangsu Province Hospital, Nanjing, China; ^4^ Department of Neurology, Jiangsu Chinese Medicine Hospital, Nanjing, China; ^5^ Department of Cardiac Surgery, The First School of Clinical Medicine, Nanjing Medical University, Nanjing, China

**Keywords:** long non-coding RNA, angiogenesis, neurodegeneration, reactive glia

## Abstract

Although nervous and vascular systems are functionally different, they usually share similar mechanisms for function maintenance. Neurovascular dysfunction has became the pathogenesis of several vascular and nervous disorders. Here we show that long non-coding RNA-MIAT is aberrantly expressed under neurovascular dysfunction condition. MIAT is shown as a regulator of vascular dysfunction, including retinal angiogenesis, corneal angiogenesis, and vascular permeability. MIAT is also shown as a regulator of retinal neurodegeneration under diabetic condition. Mechanistically, MIAT regulates neural and vascular cell function via MIAT/miR-150-5p/VEGF network. The eye is a valuable model to study central nervous system (CNS) disorders. We show that MIAT knockdown leads to cerebral microvascular degeneration, progressive neuronal loss and neurodegeneration, and behavioral deficits in a CNS neurovascular disorder, Alzheimer's disease. MIAT may represent a pharmacological target for treating neurovascular-related disorders.

## INTRODUCTION

Nervous and vascular systems are anatomically closely tied to each other. The nerves are often vascularized by vasa nervorum to supply oxygen and nutriment, whereas the vascular is often innervated by nerve fibers that regulate vascular tone. Nerves and blood vessels usually share similar mechanisms and regulators for function maintenance [1, 2]. Abnormal neurovascular interactions have been reported in several human diseases, such as stroke, brain injury, and retinopathy, and cancer [3]. Thus, clarifying the potential mechanism of neurovascular interaction would provide novel strategies for treating neurovascular dysfunction.

Long non-coding RNAs (lncRNAs) are pervasively transcribed in mammalian genome, which are defined as non-coding transcripts > 200 nucleotides. LncRNAs participate in numerous biological processes to regulate gene expression through mRNA splicing, transcription regulation, translation regulation, and genomic imprinting [4]. Abnormal lncRNA expression has been found in many human disorders ranging from neurodegeneration to cancer [5, 6]. The homeostasis and plasticity of neurovascular interaction is required for exquisite gene regulatory mechanism. Given the crucial role of lncRNAs in gene regulation, it is not surprised that lncRNAs are potential regulators of neurovascular interaction.

In previous study, we revealed a key role of lncRNA-MIAT in diabetes mellitus-induced microvascular dysfunction [7]. MIAT is expressed both in the human and mouse genome. The genomic information of MIAT in human and mouse have been reported [8]. Vascular and nervous systems are highly interactive in both physiological and pathological conditions and often share the common regulators for function maintenance [2]. We speculated that MIAT was also a critical regulator of neurovascular interaction. Here we revealed that MIAT was aberrantly expressed during neurovascular dysfunction. MIAT knockdown affected the development of vascular and neuronal degeneration. Thus, MIAT is a promising therapeutic target for treating neurovascular dysfunction-related diseases.

## RESULTS

### MIAT is aberrantly expressed during neurovascular dysfunction

**Figure 1:**
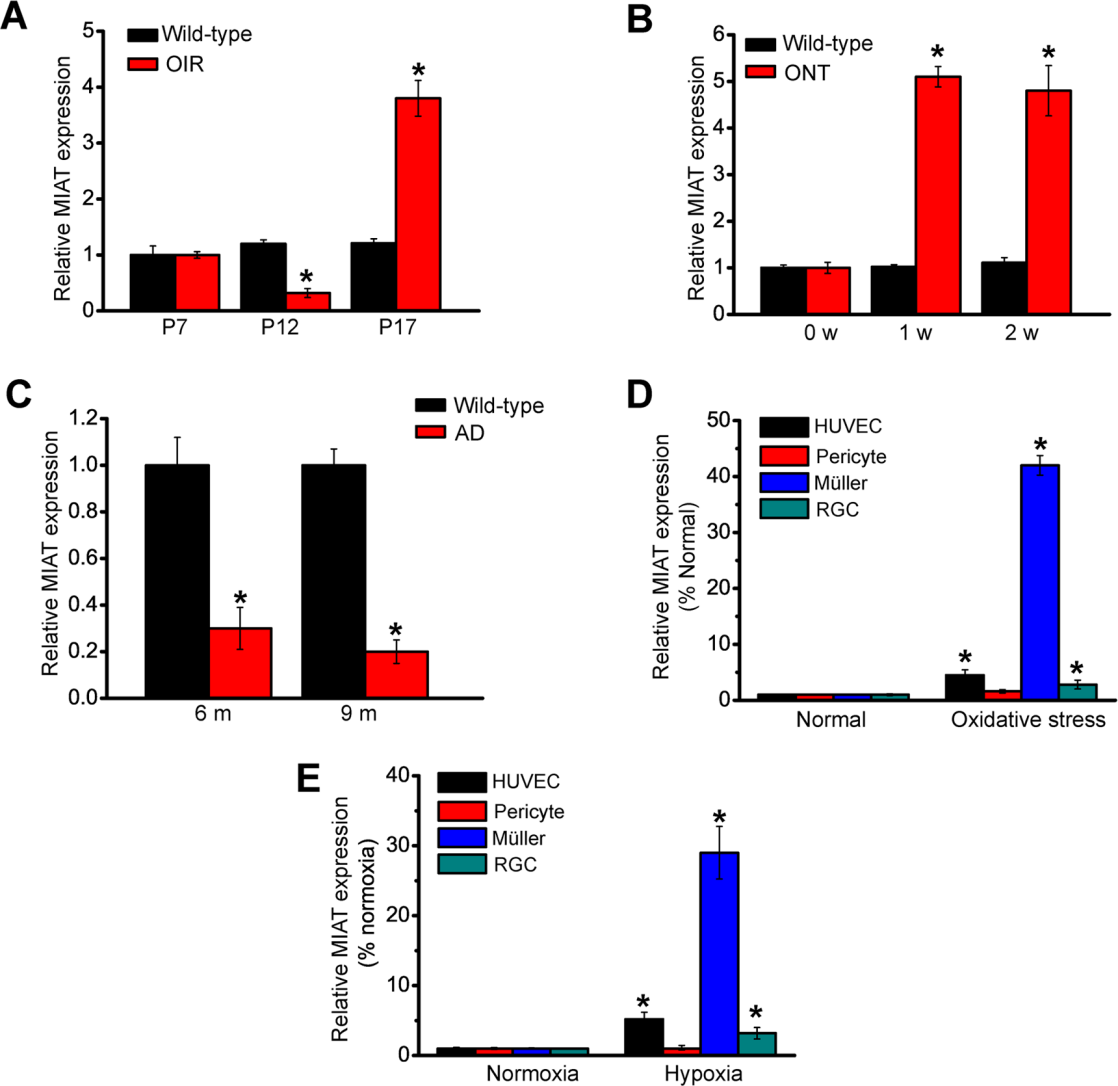
MIAT is aberrantly expressed during neurovascular dysfunction (**A**) Neonatal mice were exposed to 75% oxygen from P7 to P12, and then returned to room air. qRT-PCRs were conducted to compare MIAT expression between wild-type and OIR retinas (*n* = 6 animals per group). (**B**) qRT-PCRs were conducted to detect MIAT levels in rat retinas of 0, 1, and 2 weeks after ONT (*n* = 6 animals per group). (**C**) qRT-PCRs were conducted to compare MIAT expression in the brain parenchyma between wild-type and Alzheimer's disease transgenic mice (*n* = 6 animals per group). (**D**, **E**) Human umbilical vein endothelial cells (HUVEC), primary pericytes, primary rat retinal ganglion cells (RGCs), and retinal Müller cell line (rMC-1) was exposed to H_2_O_2_ (50 μm, *n* = 4), or hypoxia (CoCl_2_, 200 μm, *n* = 4) for 24 h. MIAT expression was detected by qRT-PCRs. *P < 0.05. All data were from three independent experiments.

We determined whether MIAT expression is altered during neurovascular dysfunction condition. In the mouse model of oxygen-induced retinopathy (OIR), MIAT expression was significantly reduced at the vaso-obliteration stage (P7-P12), whereas MIAT expression was increased at the neovascularization stage (P12-P17) (Figure 1A). In the optic nerve transection (ONT) model, MIAT expression was significantly reduced in the ONT retinas (Figure 1B). Neurovascular dysfunction is also implicated in the pathogenesis of Alzheimer's disease. MIAT expression was significantly down-regulated in the brain parenchyma of Alzheimer's disease transgenic mice (Figure 1C). Diabetic retinopathy is associated with retinal neurodegeneration and vascular dysfunction. MIAT expression was significantly up-regulated in the retinas of diabetic rats and db/db mice [7]. Collectively, these results suggest that MIAT is potentially involved in neurovascular dysfunction.

We also investigated whether MIAT expression is altered in diseased condition *in vitro*. Endothelial cells, pericytes, glia and neurons compose neurovascular unit (NVU) to regulate neurovascular crosstalk. MIAT expression was significantly up-regulated in endothelial cells, glia, and neurons in response to hypoxic or oxidative stress (Figure 1D and 1E). Notably, greatest expression change of MIAT was detected in Müller glia.

### MIAT is an important regulator of vascular remodeling

**Figure 2:**
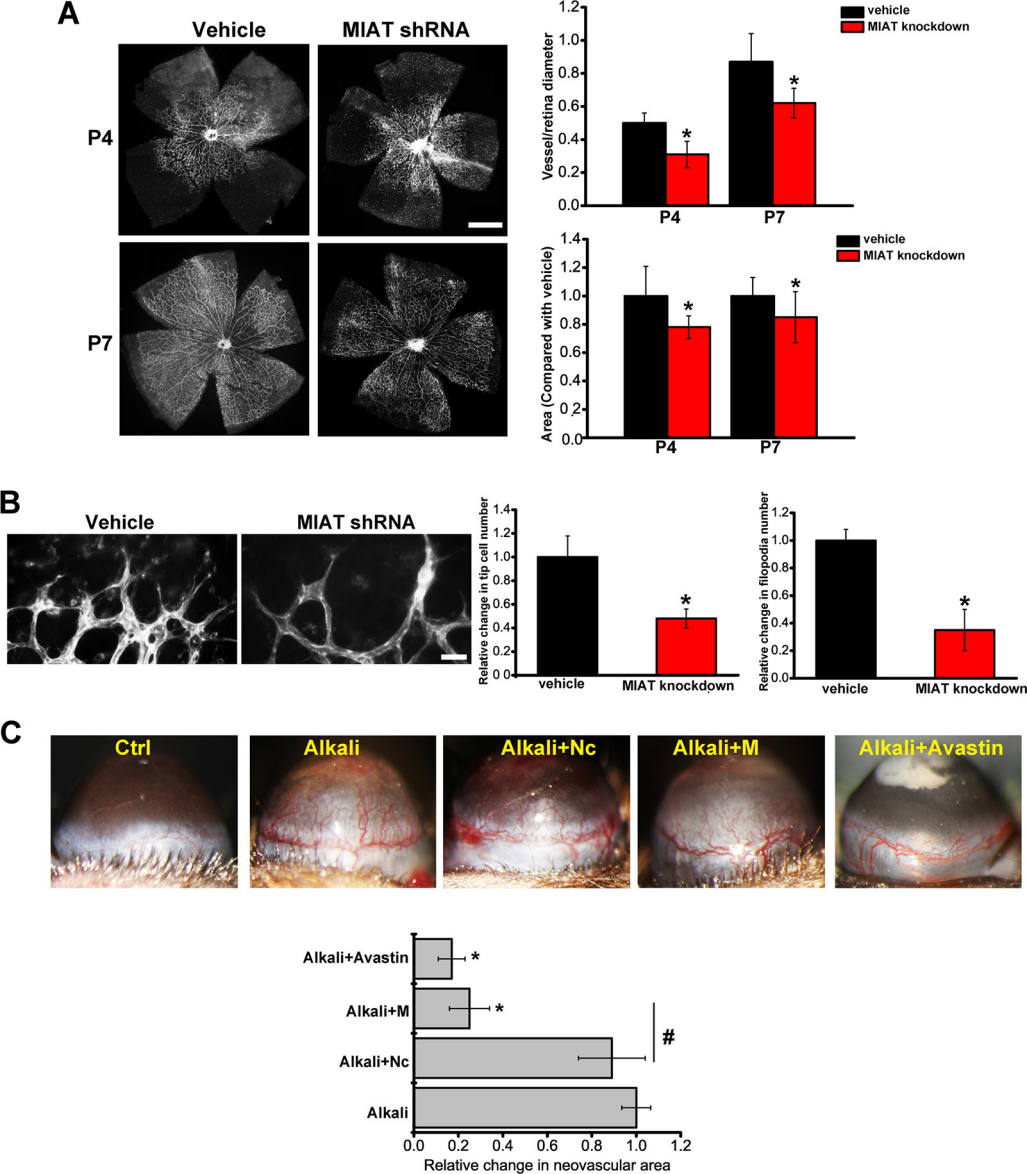
MIAT is an important regulator of vascular remodeling (**A**) The retinas of C57B/6 mice were injected with vehicle or MIAT shRNA for 4 d or 7 d. Isolectin B4 staining and quantification of vascuarization was conducted (*n* = 6 animals per group). Scale bar, 500 μm. (**B**) Isolectin B4 stained tip cells and filopodia at P7 and quantification of tip cells and filopodia number was conducted. Scale bar, 50 μm. (**C**) C57B/6 mice were received alkali-burn on the central corneas of their eyes, and treated as shown for 48 h. On day 4, corneal neovasculature was monitored by slit lamp and quantified (*n* = 6 animals per group). *P < 0.05. All data were from three independent experiments.

We employed retinal vascular development model to investigate the role of MIAT in vascular remodeling *in vivo*. MIAT knockdown displayed a significant delay in radial extension of vascular plexus from optic nerve to periphery at postnatal days 4 (P4) and P7, as well as fewer branch points (Figure 2A). MIAT knockdown retinas had fewer tip cells and filopodia (Figure 2B).

We also employed corneal neovascularization model to study the role of MIAT in vascular remodeling. Corneal neovasculature extended from the lumbus toward the pellets after alkali-burn. Notably, MIAT knockdown significantly decreased alkali-burn-induced angiogenesis, which was comparable to Avastin (anti-VEGF) treatment in C57B/6 mice (Figure 2C).

### MIAT is shown as a regulator of retinal neurodegeneration

**Figure 3:**
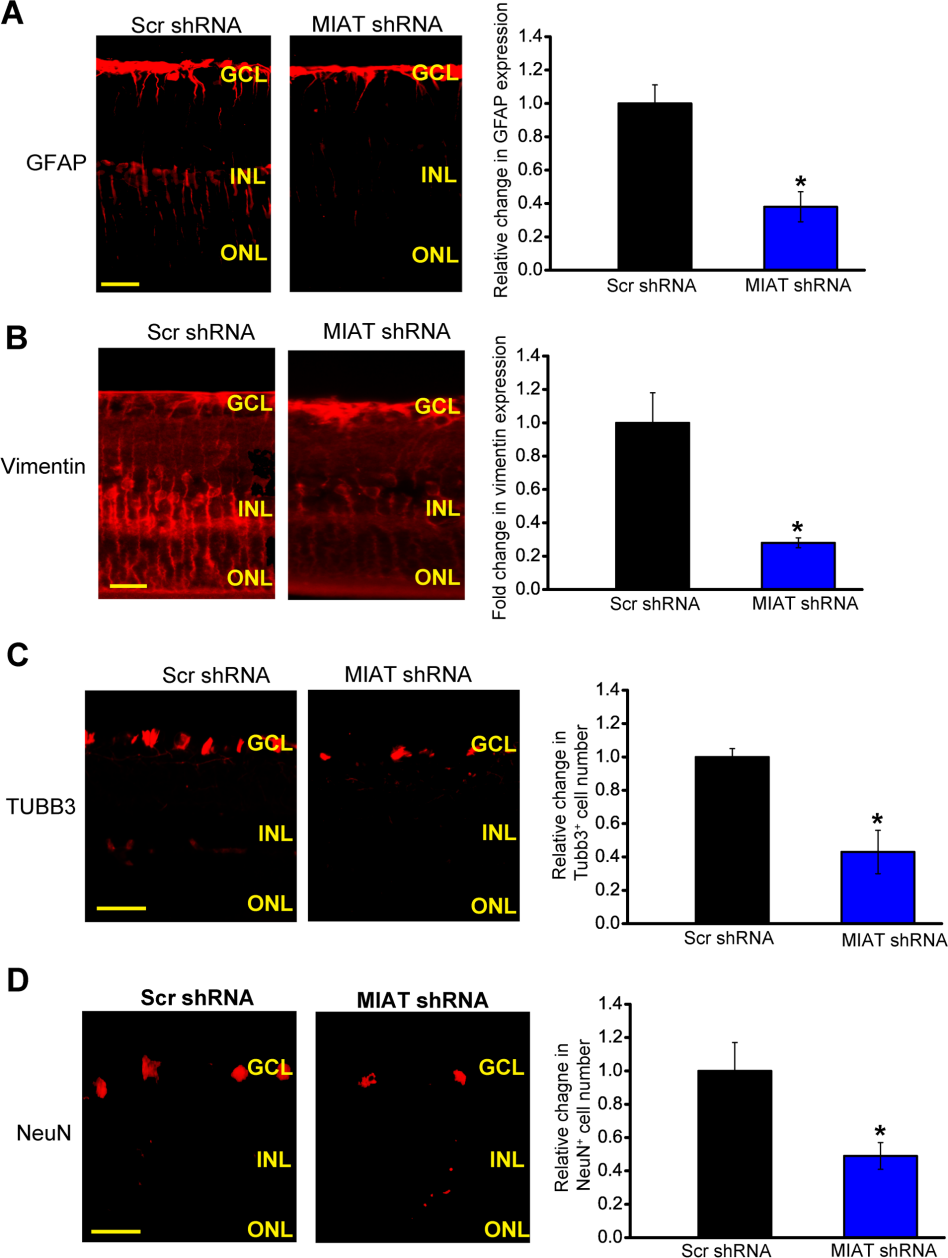
MIAT is shown as a regulator of retinal neurodegeneration (**A**–**D**) Four-month old diabetic mice were received an intravitreous injection of scrambled (scr) shRNA or MIAT shRNA viral vector for the indicated time period. Retinal slices were stained with glial fibrillary acidic protein (GFAP) (A), vimentin (B), TUBB3 (C), and NeuN (D). Quantitative analysis showed that MIAT knockdown affected retinal reactive gliosis and RGC number. Scale bar, 100 μm. GCL, ganglion cell layer; INL, inner nuclear layer; RGC, retinal ganglion cell; ONL, outer nuclear layer (*n* = 6 animals per group). *P < 0.05. All data were from three independent experiments.

Nervous and vascular systems usually share the common regulator for function maintenance. Since MIAT was shown as a regulator of vascular remodeling, we speculated that MIAT was also involved in retinal neurodegeneration. Immunofluorescence staining showed that compared with diabetic group, MIAT knockdown significantly attenuated reactive gliosis as shown by decreased glial fibrillary acidic protein (GFAP) and vimentin (markers of Müller glial cells) staining (Figure 3A and 3B). MIAT knockdown decreased the number of NeuN- or TUBB3-positive RGCs. These results indicate that MIAT regulates reactive gliosis and RGC survival (Figure 3C and 3D).

Retinal slices were also stained with other protein markers, including calretinin (ganglion cells and amacrine cells), calbindin (amacrine and horizontal cells), rhodopsin (Rod and cone photoreceptor), and protein kinase Cα (PKCα; bipolar cells). The result showed that MIAT knockdown significantly decreased calbindin-labeled cells in the GCL, but did not change calbindin-labeled cells in the INL (Figure S1). MIAT knockdown significantly decreased calbindin-labeled cells in the GCL, but did not affect calbindin-labeled horizontal and amacrine cells (Figure S1). We did not observe significant difference of rhodopsin-labeled photoreceptors and PKCα-labeled bipolar cells between the retinas with and without MIAT knockdown (Figure S1).

### MIAT regulates vascular and neural cell function *in vitro*

**Figure 4:**
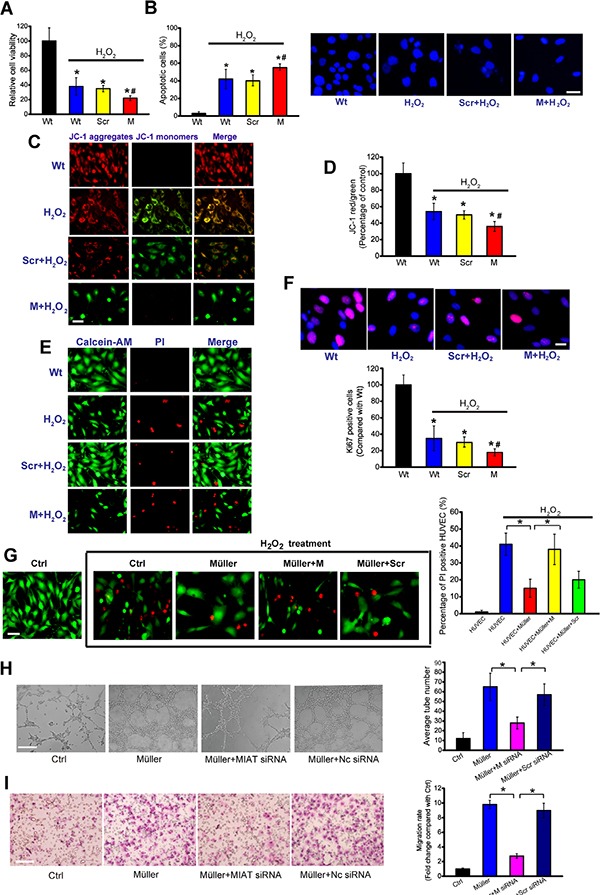
MIAT regulates the function of neurovascular unit *in vitro* (**A**) Müller cells were transfected with scrambled (Scr) siRNA, MIAT siRNA, or left untreated, and then exposed to H_2_O_2_ (50 μm) for 48 h. Cell viability was detected using MTT method (*n* = 4). (**B**) Apoptotic cells were detected using Hoechst staining and quantitated (*n* = 4). Scale bar, 20 μm. (**C**, **D**) Müller cells were incubated with JC-1 probe at 37°C for 30 min, centrifuged, washed, transferred to a 96-well plate (100,000 cells per well), observed using a fluorescence microscope (C, *n* = 4), and assayed using a fluorescence plate reader (D, *n* = 4). Scale bar, 50 μm. (**E**) Cell apoptosis was analyzed using calcein-AM/PI staining. Green: live cells, Red: dead or dying cell. Scale bar, 50 μm. (**F**) Ki67 immunofluorescence staining and quantitative analysis revealed that MIAT knockdown reduced Müller cell proliferation (*n* = 4). Scale bar, 20 μm. (**G**) HUVECs were co-cultured with Müller cells, and then treated with H_2_O_2_ (50 μm) for 48 h. PI staining and quantitative analysis was conducted to detect the dead or dying cells (*n* = 4). Scale bar, 20 μm. (**H**) HUVECs were co-cultured with Müller cells as shown. The tube-like structures were observed 24 h after cell seeding. Average number of tube formation for each field was statistically analyzed (*n* = 4). Scale bar, 100 μm. (**I**) HUVEC migration was detected using Transwell migration assay. Images were taken 24 hours after cell seeding (*n* = 4). Scale bar, 100 μm.

Vascular and neural cells interact with each other to maintain homeostatic microenvironment [8]. We have revealed that MIAT knockdown could affect endothelial cell function *in vitro* [7]. Here, we studied the functional significance of MIAT alteration in Müller cells and neurons *in vitro*. In response to oxidative stress, MIAT knockdown significantly decreased Müller cell viability (Figure 4A), and accelerated cell apoptosis as shown by increased apoptotic nuclei (condensed or fragmented) (Figure 4B), decreased mitochondrial depolarization (Figure 4C and 4D), and increased PI-positive cells (dying or dead cells) (Figure 4E). Ki67 staining showed that MIAT knockdown decreased Müller cell proliferation (Figure 4F). MIAT knockdown also decreased Müller cell viability, accelerated cell apoptosis, and reduced cell proliferation upon hypoxia stress (Figure S2). We then investigate the effect of MIAT knockdown on RGC function. MIAT knockdown decreased RGC cell viability and accelerated RGC apoptosis upon oxidative stress and hypoxia stress (Figure S3 and S4).

Upon oxidative or hypoxic stress, greatest expression change of MIAT was detected in Müller cells, compared with neurons and endothelial cells (Figure 1D and 1E). We thus speculated that MIAT expressed in Müller cells played a prominent role in neurovascular interaction. PI staining revealed that Müller cell co-culture significantly decreased the number of apoptotic RGCs and endothelial cells induced by hypoxic or oxidative stress. MIAT knockdown in Müller cells significantly reduced the protective effect (Figures 4G and S5). We also revealed that MIAT knockdown in Müller cells significantly reduced endothelial cell migration and tube formation (Figure 4H and 4I).

### MIAT regulates the production of neurotrophic and angiogenic factors

**Figure 5:**
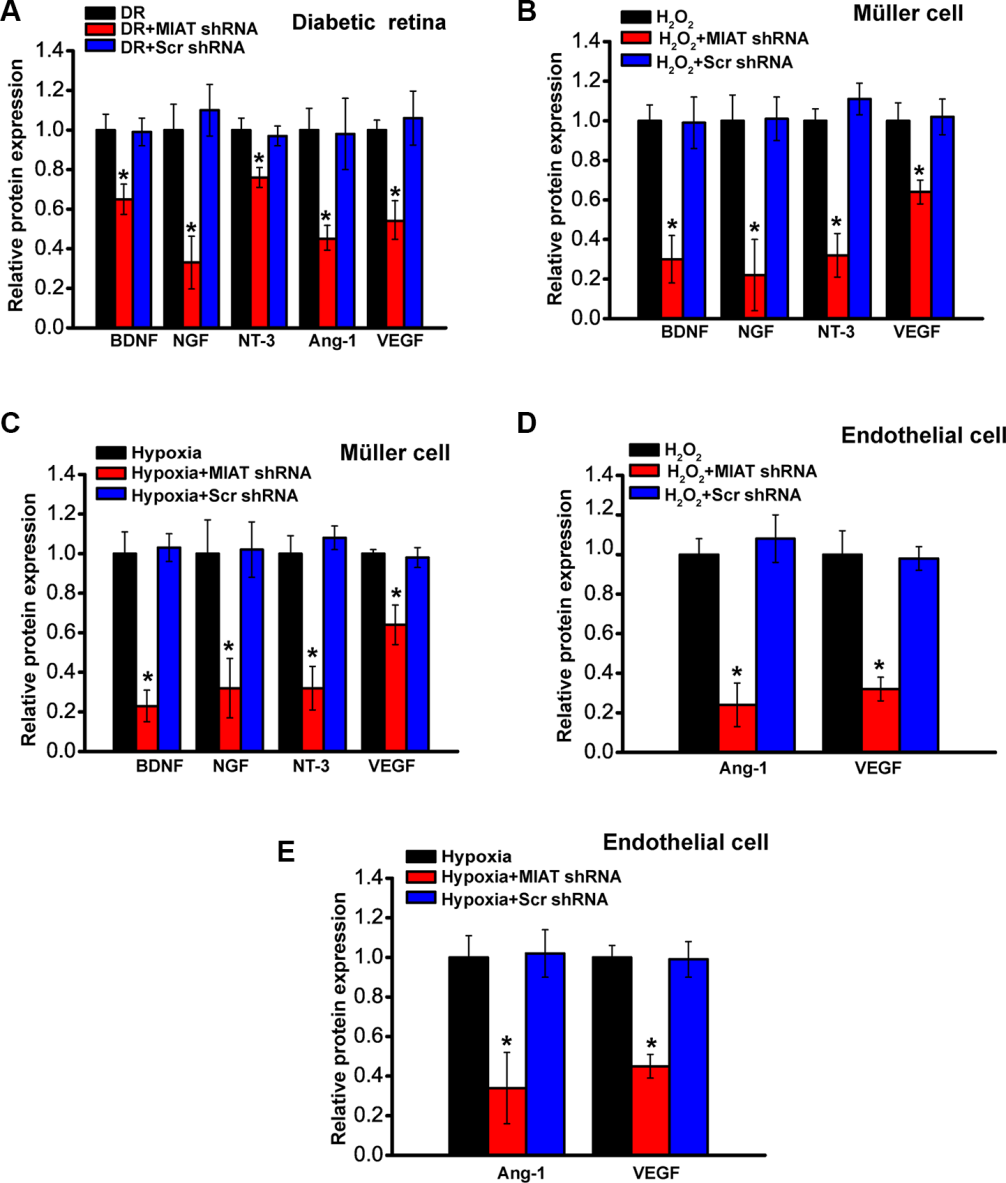
MIAT regulates the production of neurotrophic and angiogenic factors (**A**) Diabetic mice were received an intravitreous injection of scrambled (scr) shRNA or MIAT shRNA viral vector, or left untreated for four months. (**B**, **C**) Müller cells were transfected with scr siRNA, MIAT siRNA, or left untreated, and then exposed to H_2_O_2_ (50 μm) or hypoxia (CoCl_2_, 200 μm) for 48 h. (**D**, **E**) Endothelial cells were transfected with scr siRNA, MIAT siRNA, or left untreated, and then exposed to H_2_O_2_ (50 μm) or hypoxia (CoCl_2_, 200 μm) for 48 h. Effect of MIAT knockdown on the production of neurotrophic and angiogenic factors was determined (*n* = 6). *P < 0.05. All data were from three independent experiments. GNDF, glial cell line-derived neurotrophic factor; NT-3, neurotrophin-3; NGF, nerve growth factor; BDNF, brain-derived neurotrophic factor; VEGF, vascular endothelial growth factor; ANG-1, Angiopoietin-1.

The above-mentioned results indicate that MIAT regulates neurovascular interaction *in vivo* and *in vitro*. We then investigated whether MIAT knockdown affected the production of neurotrophic factors and angiogenic factors due to their critical roles in neurovascular regulation. MIAT knockdown decreased the production of BDNF, NGF, NT-3, Ang-1, and VEGF in diabetic retinas (Figure 5A). Under oxidative stress or hypoxic stress, MIAT knockdown decreased the production of BDNF, NGF, NT-3, and Ang-1 in Müller cells (Figure 5B and 5C). Moreover, MIAT inhibitory effect on Ang-1 or VEGF production was observed in endothelial cells (Figure 5D and 5E). Collectively, these results indicate that MIAT knockdown affects the production of neurotrophic and angiogenic factors.

### MIAT/miR-150-5p/VEGF network is involved in neurovascular dysfunction

**Figure 6:**
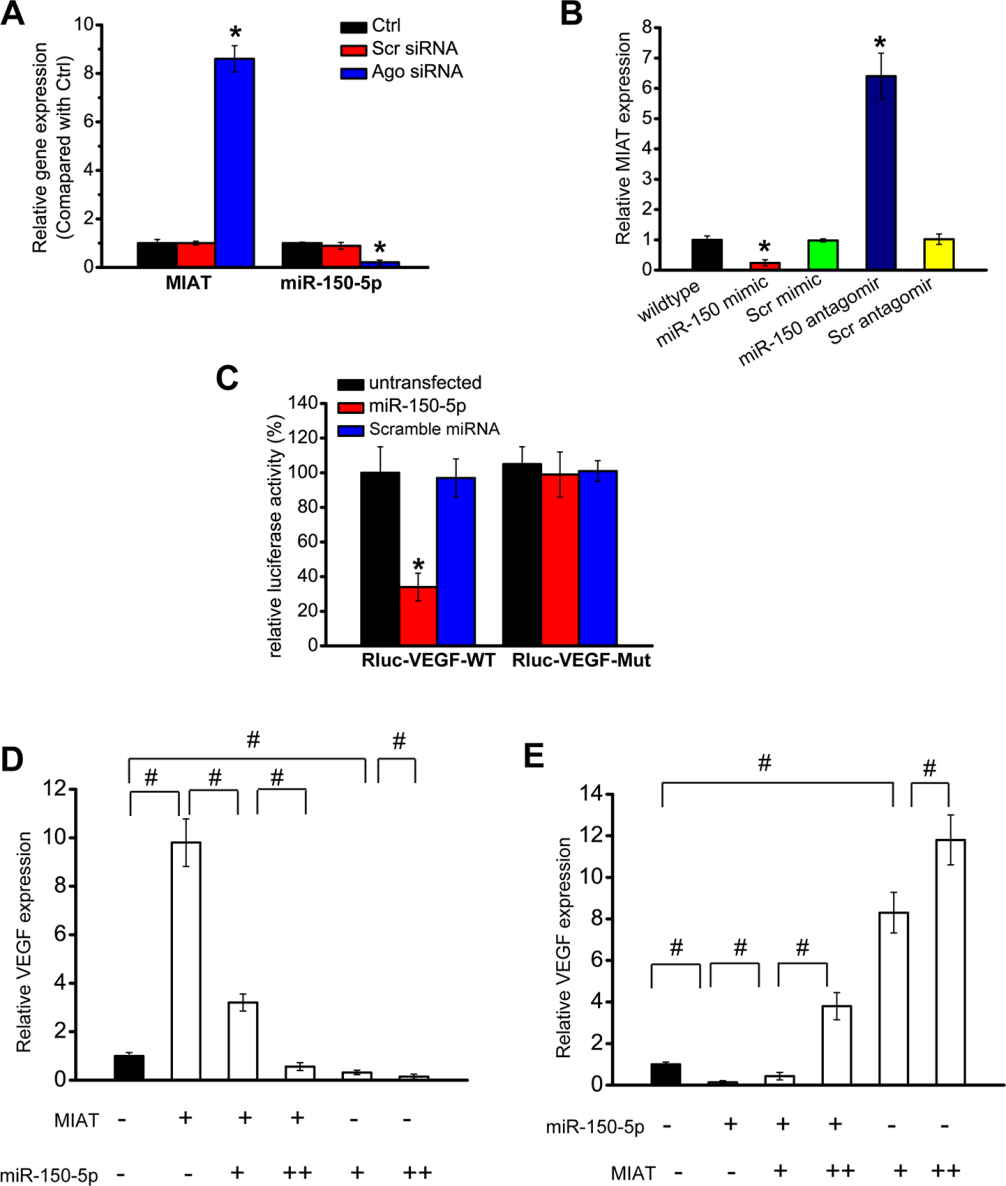
MIAT/miR-150-5p/VEGF constitutes a regulatory network (**A**) Müller cells were transfected with Ago2 siRNA, scrambled siRNA, or left untreated. miR-150-5p and MIAT levels were detected using qRT-PCRs. (**B**) Müller cells were transfected with miR-150 mimic, scrambled mimic (Scr), miR-150 antagomir, scrambled antagomir (Scr) or left untreated. miR-150-5p or MIAT levels were detected using qRT-PCRs. (**C**) VEGF (RLuc-VEGF-WT) and mutant (RLuc-VEGF-Mut) were cloned into the downstream of luciferase vector. Luciferase activity was detected using the dual luciferase assay. (**D**, **E**) Müller cells were transfected with different combinations of MIAT and miR-150-5p mimic. qRT-PCRs were conducted to detect VEGF expression. (+) corresponds to 100 ng MIAT construct or 20 ng of miR-150-5p mimic. (++) corresponds to 200 ng MIAT construct or 50 ng of miR-150-5p mimic. “^*^” indicated significant difference compared with the corresponding control group. “^#^” indicated significant difference between the marked groups.

MIAT/miR-150-5p/VEGF network has been reported to regulate endothelial cell function [7]. We speculated that MIAT/miR-150-5p/VEGF network can also regulate neurovascular interaction by targeting other cells, especially Müller glia. Ago2 is [9]. We thus investigated whether MIAT expression is under the control of miRNAs by knocking down Ago2 in Müller cells. Increased MIAT expression was detected in Ago2 knockdown cells, whereas miR-150-5p stability was impaired by Ago2 knockdown (Figure 6A). We then determined whether miR-150-5p directly regulates MIAT in Müller cells. miR-150-5p mimic obviously reduced MIAT expression, whereas miR-150-5p antagomir obviously up-regulated MIAT expression in Müller cells (Figure 6B). miR-150-5p up-regulation significantly decreased the viability and proliferation of Müller cells, whereas MIAT overexpression partially abrogated miR-150-5p repression effect (Figure S6).

We also investigated whether miR-150-5p regulates VEGF expression in Müller cells. Luciferase assay revealed that miR-150-5p directly regulated VEGF expression in Müller cells (Figure 6C). We further determined whether the MIAT-VEGF cross-talk is involved in regulating Müller cell function. MIAT knockdown significantly inhibited the viability and proliferation of Müller cells, whereas exogenous VEGF treatment could partially abrogate this inhibition (Figure S7). These results suggest that MIAT-VEGF crosstalk regulates Müller cell function.

If MIAT functions as a decoy, the relative concentration of MIAT and miR-150-5p could affect VEGF expression. miR-150-5p was gradually increased in the presence or absence of MIAT. We showed that MIAT overexpression significantly increased VEGF level, and was reduced when miR-150-5p level was up-regulated (Figure 6D). MIAT amount was also gradually increased in the presence or absence of miR-150-5p. miR-150-5p overexpression significantly reduced VEGF level, whereas reduction was reversed when MIAT level was increased (Figure 6E). These results indicate that there is interplay among MIAT, miR-150-5p, and VEGF in Müller cells.

### MIAT reduction exacerbates vasculo-neuronal dysfunction in Alzheimer's disease

**Figure 7:**
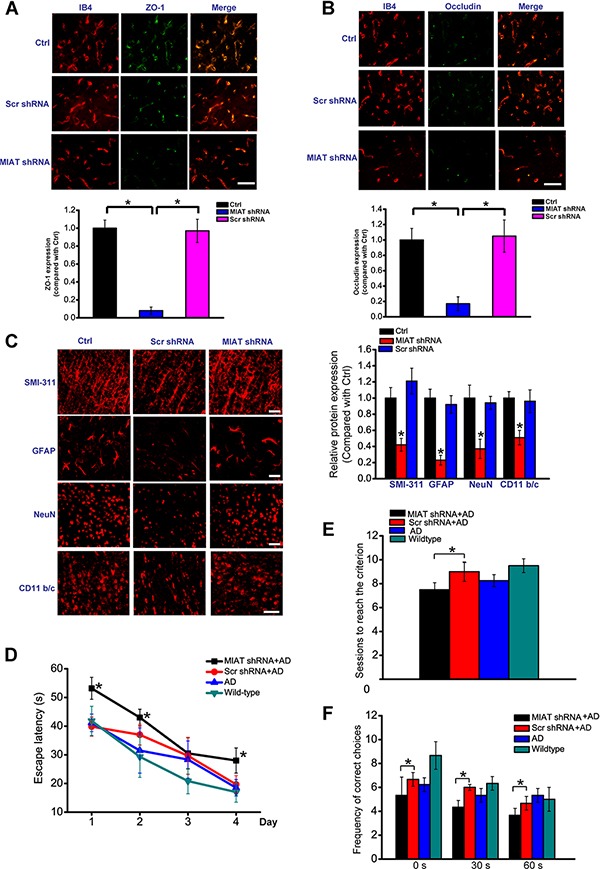
MIAT reduction exacerbates Alzheimer's disease vasculo-neuronal dysfunction (**A**, **B**) ZO-1 or occludin (green) and IB4-positive capillary profiles (red) and quantification of endothelial occludin and ZO-1 expression in the hippocampus of 12-month-old APP/PSN mice injected with MIAT shRNA, scr shRNA, or left untreated. scale bar, 20 μm. (**C**) Immunostaining and quantification of SMI-311, NeuN, GFAP, and CD11b/c in the CA1 hippocampal regions of APP/PSN mice injected with MIAT shRNA, Scr shRNA, or left untreated at the 12-month-old showed accelerated neurodegenerative changes in MIAT-knockdown mice. scale bar, 20 μm. (**D**) Intrahippocampal injection of MIAT shRNA exacerbated learning deficits in APP/PS1 mice as shown by increased escape latency. (**E**) The ability of animals to learn spatial alternation was tested using T-maze apparatus. The number of sessions mice needed to reach the learning criterion in the spatial alternation task was indicated. (**F**) The number of correct choices in the spatial delayed alternation test wass indicated. The correct choice in the delays of 30 and 60 s was significantly reduced in MIAT shRNA-injected AD mice compared with scr shRNA-injected AD mice.

We also determined the role of MIAT in vasculo-neuronal dysfunction in Alzheimer's disease. Intrahippocampal injection of MIAT shRNA was conducted to down-regulate MIAT levels. Immunostaining for tight junction proteins, occludin and zonula occludens-1 (ZO-1), each combined with isolectin B4 staining, indicated that MIAT knockdown significantly decreased brain microvessel number and the expression of tight junction proteins (Figure 7A and 7B).

We further determined the effect of MIAT knockdown on neurodegenerative change in APP/PSN mice. SMI-311 immunostaining was conducted to show neuritis, and 42% reductions in hippocampal SMI-311 positive neurofilaments were detected in the hippocampus of MIAT knockdown mice (Figure 7C). MIAT knockdown led to a marked increase in neuronal loss as shown by decreased NeuN-positive cells (Figure 7C). We also detected astrocytosis using GFAP as an astrocytic marker and microgliosis using CD11b/c as a microglial marker. MIAT knockdown significantly reduced the up-regulation of GFAP and CD11b/c immunoreactivity in the hippocampus (Figure 7C).

Neurovascular dysfunction exacerbates AD pathogenesis by affecting Aβ clearance and increasing brain Aβ levels [11]. We showed that MIAT knockdown increased Aβ40 and Aβ42 levels, accelerated and exacerbated cerebral β-amyloidosis as shown by Aβ immunostaining. Low-density lipoprotein receptor related protein 1 (LRP1) is a key Aβ clearance transporter at the blood-brain barrier (BBB). Western blots revealed a significant reduction in brain LRP1 in MIAT knockdown mice (Figure S8).

To investigate whether MIAT knockdown affects the learning and memory impairment in APP/PSN mice, MIAT shRNA was injected into the hippocampus at the age of 8 months when full-blown amyloid plaque pathology had already developed. Spatial learning experiment revealed that MIAT knockdown mice showed a significant impairment in spatial learning ability as shown by longer time of escape latency compared with AD or AD+Scr shRNA injected mice (Figure 7D). We next tested the ability of animals to learn spatial alternation using T-maze apparatus to gain a reward. A significant statistical difference in the number of sessions to reach the learning criterion was detected between MIAT shRNA-injected and Scr shRNA-injected group (Figure 7E). MIAT shRNA-injected group showed decreased spatial delayed alternation (Figure 7F). These results indicate that MIAT knockdown exacerbates learning and memory impairment in APP/PSN mice.

## DISCUSSION

Blood vessels and nerves are functionally interdependent. Dysfunction of their cross-talk has become the pathogenesis of several human disorders [9]. Here we showed that lncRNA-MIAT was involved in the maintenance of proper microvascular and nervous function. MIAT regulated the function of neural and vascular cells through MIAT/miR-150-5p/VEGF network. MIAT knockdown also led to cerebral microvascular degeneration, progressive neurodegeneration, and behavioral deficits, suggesting a crucial role of MIAT in the pathogenesis of neurovascular dysfunction.

MIAT is widely expressed in endothelial cells, Müller glia, and neurons. Its knockdown could affect the viability, proliferation, and apoptosis of these cells [7]. Müller cells are the major glia of the retina [12, 13]. Upon stress, Müller cells had the greatest change of MIAT expression, implying a prominent role of MIAT in Müller cells. *In vitro* co-culture system study showed that MIAT knockdown in Müller cells decreased RGC survival, endothelial cell survival, endothelial cell tube formation and migration, implying that Müller glial dysfunction may be the upstream cause of retinal neuronal and vascular dysfunction.

We previously reveal that MIAT functions as a ceRNA to regulate VEGF levels by sponging miR-150-5p in retinal endothelial cells. MIAT overexpression is a sink for miR-150-5p, thereby affecting the derepression of VEGF [7]. Here we found a similar regulatory mechanism in Müller cells. VEGF can be released from Müller cells under stress condition, such as hypoxic condition [14–16]. VEGF supports the survival of retinal endothelial cells and neurons, and restricts glucose- or oxidative stress-induced injuries. The protective effects of VEGF include vasodilation, glial cell proliferation, inflammation, and neuroprotection [17, 18]. MIAT overexpression released miR-150-5p repressive effect on VEGF. This regulatory loop maintained a relative balance for glia function, and indirectly regulated RGC and endothelial cell function, to resist external stress.

The eye is an extension of the brain. It displays several similarities to the brain in terms of anatomy, functionality, and stress tolerance [19]. Some neurodegenerative processes in CNS disorders can be detected in some ocular pathology [20]. We showed that MIAT was implicated in the vasculo-neuronal dysfunction and degeneration in Alzheimer's disease. MIAT knockdown led to endothelial cell injury and cerebral microvascular dysfunction. The delivery of oxygen and nutrients to other part of the body could be affect, which in turn lead to neuronal dysfunction, such as the retraction of neurites, neuronal loss and substantial behavioral deficit. Neuronal dysfunction in MIAT knockdown mice likely reflected synergistic effects of MIAT-mediated vascular injury, diminished neuronal glucose delivery and greater accumulation of Aβ neurotoxic species.

The interactions among multiple cells including neuronal, glial, and vascular cells, are critical for maintaining adequate blood flow that is necessary for normal tissue function. Neurovascular dysfunction would become the pathogenesis of human diseases, such as retinopathy, stroke and Alzheimer's diseases [21]. Our findings demonstrate that MIAT is a critical regulator of vascular integrity and neuronal function. MIAT may represent a therapeutic target for treating neurovascular injury and the resulting neuronal dysfunction.

## MATERIALS AND METHODS

### Animal experiment

This study was approved by the Nanjing Medical University Animal Care and Use Committee, and complies with the Guide for the Care and Use of Animals published by the National Institutes of Health. Experiments were reported according to the ARRIVE guidelines. All experiments were also conducted according to the ARVO statement for the use of animals ophthalmic and vision research. Experiments were performed on anesthetized and temperature controlled animals.

### Induction of diabetic mice

After a 24 h fast, four-month old male C57Bl/6J mice were injected with streptozotocin (STZ, Sigma, 60 mg/kg, 10 mM citrate buffer, pH 4.5) to induce diabetes. The non-diabetic controls received equivalent amount of citrate buffer alone. Seven days after STZ injection, animals with blood glucose levels > 16.7 mmol/L were included in diabetic group [22].

### Alkaline burn-induced corneal neovascularization

Four-month old male C57BL/6 mice were anesthetized with isoflurane (4% vol/vol) followed by topical application of 0.1% proparacaine on the cornea. A 2.5 mm diameter filter paper soaked with 0.1 M NaOH was placed on the central cornea for 30 s, followed by immediate rinsing with 0.9% saline solution for 10 s. Areas of corneal neovascularization were determined using Image J software package The neovascular area was determined by subtracting non-stimulated vascular area from vascular area [23].

### Spatial learning and memory test

The effect of MIAT knockdown on spatial learning and memory was assessed by water maze [24, 25]. Briefly, mice had 3 day of pre-training to climb the escape platform after which they underwent a 4-day testing phase with a submerged platform (10 × 10 cm) in a black pool filled with water. During the procedure, the platform location was kept constant, and the starting points were changed between four constant locations. The acquisition phase (days 1–4) consisted of 4 trials in which the mice had a maximum of 60 s to find the platform. If the animal did not find the platform during a period of 60 s, it was gently guided to it. The animal was allowed to remain on the platform for 30 s and then moved to the next initial position without leaving the tank. The time to find the platform was recorded.

### Spatial alternation training and delay test

Spatial alternation training and delay test were performed in the T-maze apparatus [24]. Each daily session consisted of 10 trials. On the first trial of each session, both goals were baited with peanuts. For the next 9 trials, the reward was placed in the arm opposite to that chosen by the mouse on the previous trial. The criterion for successful completion of training was defined as 80% correct turns averaged over 2 consecutive days. Once the criterion was reached, spatial delayed alternation was tested by interposing 30- or 60-s delay between trials. Each delay was used for 2 consecutive days.

### Statistical analysis

All data was presented as means ± SEM. All experiments were repeated at least three times. Comparison of two experimental groups was evaluated by the unpaired Student's *t*-test. Comparison of three experimental groups was evaluated by Tukey-Kramer's test after one-way ANOVA or two-way ANOVA. *P* < 0.05 was considered to be statistically significant.

## SUPPLEMENTARY MATERIALS FIGURES


