# Supplementary material for: Long non-coding RNA-MIAT promotes neurovascular remodeling in the eye and brain
Source: Oncotarget. 2016 Jul 6;7(31):49688–98. doi: 10.18632/oncotarget.10434 (PMC5226539; doi:10.18632/oncotarget.10434)
Supplement: Supplementary file 1 [file oncotarget-07-49688-s001.pdf]

Long non-coding RNA-MIAT promotes neurovascular remodeling in the eye and brain

Supplementary Materials

Western blot

Cells or tissues were lysed in RIPA protein lysis buffer. Protein concentration was determined using bicinchoninic acid assay (Pierce). About 50 µg protein was used for immunoprecipitation or was loaded per lane, subjected to SDS-PAGE, transferred onto PVDF membranes, and then blotted with specific antibody.

Müller cell and endothelial cell or RGC co-culture

1×10^6 endothelial cells or primary RGCs were seeded on 6-well plates coated with fibronectin. After 6 h, the non-adherent cells were removed and cell medium was replaced with DMEM/F12 containing 10% FBS. Transwell inserts (Millipore) were added to the wells and seeded with 5×10^5 Müller cells. After the required treatment, the insert was removed, and cell function was determined.

Viral transduction in mouse Hippocampus

Mice were anesthetized intraperitoneally with ketamine/xylazine (90 mg/10 mg/kg). Viral vectors were diluted with sterile PBS (pH 7.4) to achieve a titer of 3×10^9. MIAT shRNA or scrambled shRNA was injected bilaterally into the dentate gyrus region of the hippocampus using the coordinates: ± 3.2 mm medial/later, −2.7 mm anterior/posterior, −2.7 mm dorsal/ventral from the bregma. The preparation was injected with a speed of 0.5 µL/min over a period of 4 min using a Hamilton 5-µL syringe. The mice were allowed to recover in a heated chamber before waking.

RNA extraction and real-time PCR

Total RNAs were extracted using TRIzol reagent (Invitrogen) and then reversely transcribed using PrimeScript RT reagent Kit (TaKaRa, Dalian, China). Real-time PCRs were performed using the PikoReal™ Real-Time PCR System (Thermo Scientific). The reaction mixture (20 µL) contained 10 ng cDNA template, 200 nM each of sense and antisense primers, and 10 µL 2 × SYBR-Green PCR Mix (TaKaRa, Dalian, China). Real-time PCRs were performed in duplicate for each sample.

Hoechst staining

After the required treatment, cells were fixed with 4% formaldehyde for 15 min at room temperature, and then permeabilized using Triton-X 100 for 10 min. After three times wash, these cells were stained with Hoechst 33342 (100 µg/ml) for 10 min, and then washed with PBS buffer. Stained nuclei were observed using an Olympus IX-73 microscope.

MTT assay

Cell viability was detected using 3-(4, 5-dimethylthiazol-2-yl)-2, 5- diphenyl-tetrazolium-bromide assay (MTT). Briefly, cells were plated at a density of 1×10^4 cells per well in 96-well plates. After the required treatment, they were incubated with MTT (0.5 mg/ml) at 37°C for 3 h. Finally, DMSO solution (100 mM) was added to dissolve formazan crystals. The absorbance was detected at 570 nm wavelength using a microplate reader (Molecular Devices).

Mitochondrial membrane potential detection

The cells (2×10^6 cells/ml) were incubated with the fluorescent dye, JC-1 (Molecular Probes), at 37°C for 3 h to detect mitochondrial membrane potential (Δψm). The fluorescence intensity was detected using a microplate reader (Molecular Devices) (excitation at 485 nm, emission spectra between 530–620 nm). An intact Δψm allows JC-1 having a delocalized positive charge, which shows red fluorescence, whereas Δψm collapse allows JC-1 dye to remain in the cytoplasm showing green fluorescence in apoptotic cells. Mitochondrial depolarization was expressed as the decrease in the red-to-green fluorescence intensity ratio.

Luciferase assay

The 3′-untranslated regions (UTR) of VEGF was cloned into the downstream of the firefly luciferase gene in pGL3 vector (Promega). The 3′-UTR without miR-150-5p binding site was constructed to generate pGL3-VEGF mutant. For luciferase assays, cells were pre-plated in 24-well plates, and then transfected with either wild-type or mutant construct with and without miRNA mimic or negative control mimic. Luciferase activities were detected using a Dual Luciferase Reporter Assay System (Promega).

Calcein-AM and propidium iodide (PI) double staining

Calcein-AM and PI double staining was used to detect live and dead cells. After the required treatment, cells were fixed with 4% formaldehyde for 15 min, and then stained using Calcein-AM solution (10 µmol/L) for 10 min. After

washing with PBS, these cells were stained with PI solution (10 µmol/L) for 10 min. The live cells were observed using a 490 nm excitation filter, whereas the dead cells were observed using a 545 nm excitation filter.

Transwell cell migration assay

Endothelial cells were seeded onto the upper chamber of 12-well Transwell plates. Medium containing 10% FBS was placed in the lower chamber and served as a chemoattractant. After the required treatment, the cells on the upper surface of the filter were removed by gently wiping with a cotton swab after 24 h treatment. The migrated were fixed and stained with crystal violet.

Intravitreal injection

The mice were anesthetized with 2% isoflurane, and a drop of 0.5% proparacaine was administered as a topical local anesthesia. A fine glass micropipette connected to a 10 µl Hamilton glass syringe was inserted through the incision in the cornea and slid between the iris and the lens into the posterior chamber of the eye. Each eye received about 3 µl adenovirus. Injections were administered slowly over approximately one minute and monitored with a stereo microscope.

Immunohistochemistry

The eyes of diabetic mice were removed, fixed in 4% PFA at 4°C for 12 h. Eyes were then cryoprotected in 30% sucrose for 12 h, embedded in OCT medium. For immunohistochemistry, sections were permeabilised with 0.2% Triton-X for 30 min, and then blocked with 10% bovine serum albumin (BSA) for 1 h. Retinal sections were incubated with the primary antibody, including GFAP (1:200, Abcam), vimentin (1:200, Abcam), NeuN (1:100, Abcam), TUBB3(1:100, Abcam), calretinin (1:500, Chemicon), calbindin (1:200, Abcam), rhodopsin (1:1,000, Sigma), and protein kinase Cα (PKCα, 1:200, Abcam), at 4°C for 24 h. The sections were washed with PBS, and then incubated with fluorophore-conjugated secondary antibodies (Invitrogen) at 4°C for 24 h. Brains were immersion-fixed in 4% PFA at 4°C for 24 h. After cryoprotection, the hemisphere was frozen in liquid nitrogen and cut in 20 µm cryosections with an interval of 400 µm, post-fixed in acetone, and incubated with the primary antibody at 4°C for 24 h followed by fluorophore-conjugated secondary antibodies. Vascular change was examined using the tight junction proteins, occluding (Abcam, 1:100) and zonula occludens-1 (ZO-1, Abcam, 1:100), and isolectin B4 (Abcam, 1:200). Neurodegenerative changes were shown by the immunodetection of SMI-311 (1:100, Abcam), NeuN (1:100, Abcam), GFAP (1:100, Abcam), and CD11b/c (1:100, Abcam).

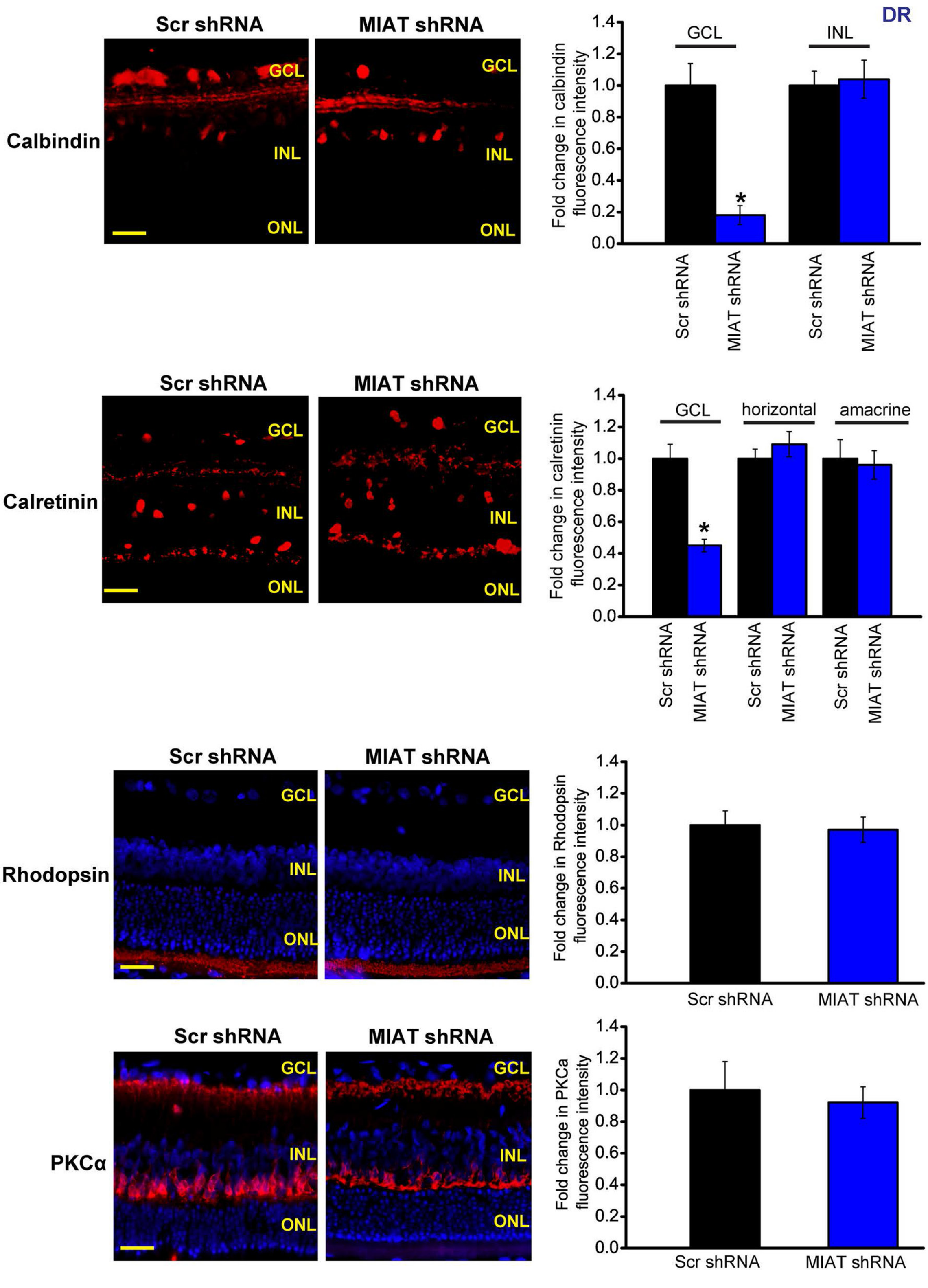

Supplementary Figure S1: MIAT knockdown does not affect retinal horizontal cells, amacrine cells, photoreceptors, and bipolar cells in diabetic retinas. Diabetic retinal slices were immunolabeled for protein markers, including rhodopsin, PKCα, calbindin, and calretinin. Quantitative analysis showed that MIAT knockdown did not affect retinal horizontal cells, amacrine cells, photoreceptors, and bipolar cells. Scale bar, 100 μm. GCL, ganglion cell layer; INL, inner nuclear layer; RGC, retinal ganglion cell; ONL, outer nuclear layer (*n* = 6 animals per group). **P* < 0.05. All data were from three independent experiments.

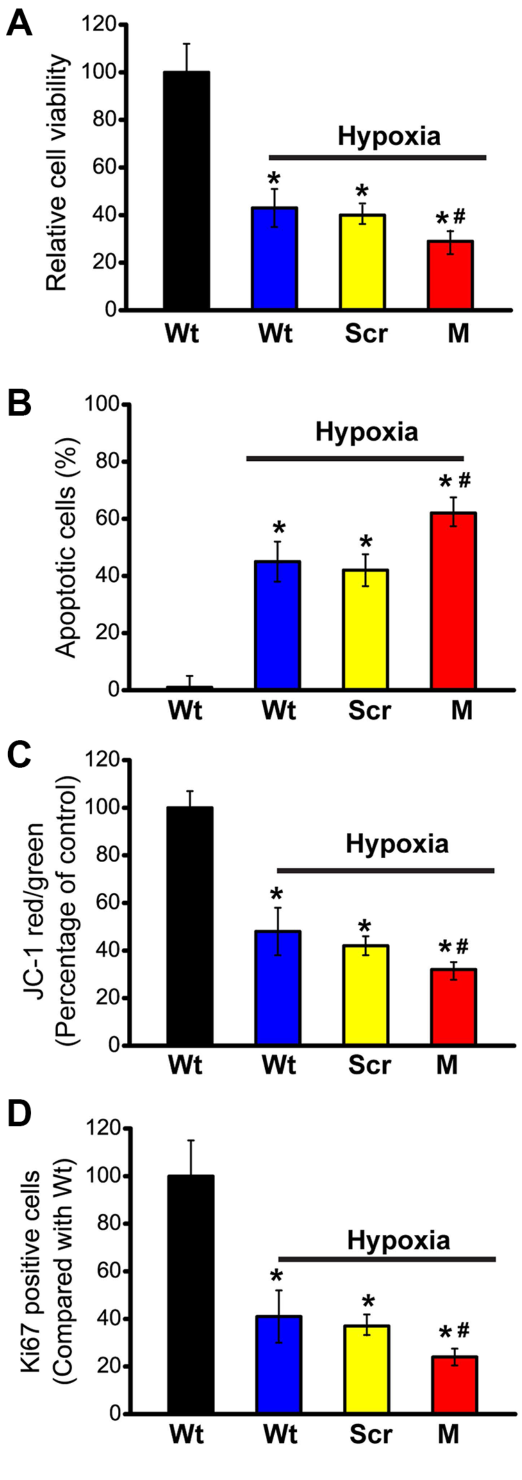

Supplementary Figure S2: MIAT regulates Müller cell function under hypoxia condition. (**A**) Müller cells were transfected with scrambled siRNA (Scr), MIAT siRNA, or left untreated, and then exposed to hypoxia (CoCl2, 200 μm) for 48 h. Cell viability was detected using MTT method ($n = 4$). (**B**) Apoptotic cells were detected using Hoechst staining and quantitated ($n = 4$). (**C**) Müller cells were incubated with JC-1 probe at 37 °C for 30 min, centrifuged, washed, transferred to a 96-well plate (100 000 cells per well), and assayed using a fluorescence plate reader ($n = 4$). (**D**) Ki67 immunofluorescence staining and quantitative analysis revealed that MIAT knockdown reduced Müller cell proliferation ($n = 4$).

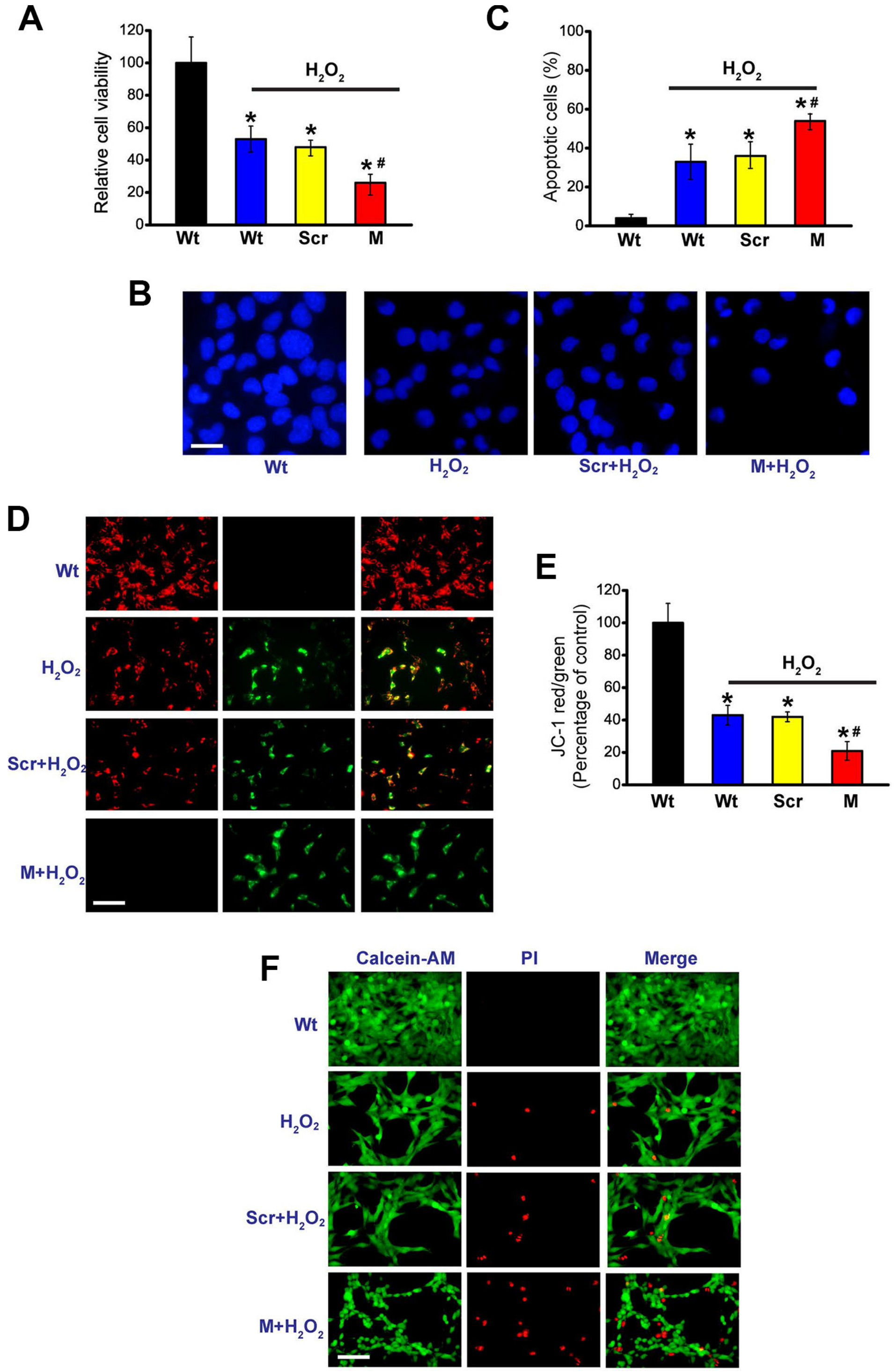

Supplementary Figure S3: MIAT regulates RGC function upon oxidative stress. (**A**) RGCs were transfected with scrambled siRNA (Scr), MIAT siRNA, or left untreated, and then exposed to H_2O_2 (50 μm) for 48 h. Cell viability was detected using MTT method (*n* = 4). (**B**, **C**) Apoptotic cells were detected using Hoechst staining and quantitated (*n* = 4). Scale bar, 20 μm. (**D**, **E**) RGCs were incubated with JC-1 probe at 37 °C for 30 min, centrifuged, washed, transferred to a 96-well plate (100 000 cells per well), observed using a fluorescence microscope (D, n=4), and assayed using a fluorescence plate reader (E, *n* = 4). Scale bar, 50 μm. (**F**) Dead or dying cells were analyzed using calcein-AM/PI staining. Green: live cells, Red: dead or dying cell (*n* = 4). Scale bar, 50 μm.

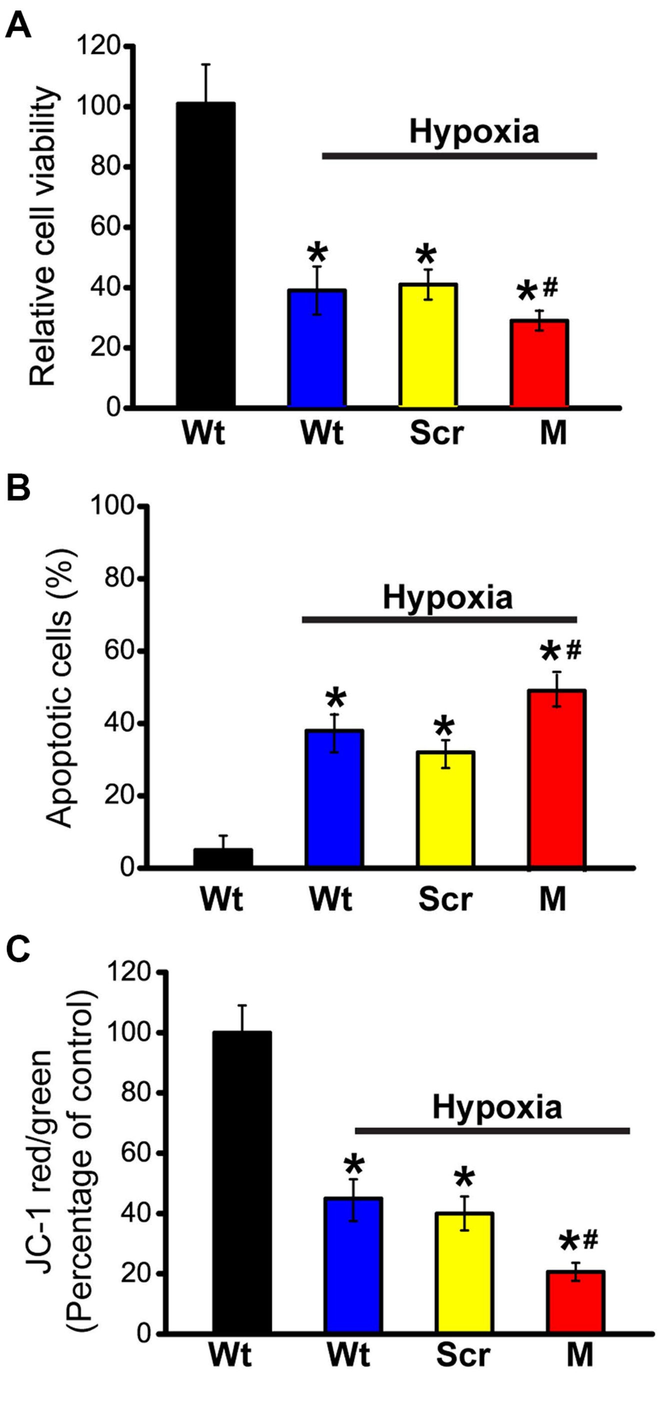

Supplementary Figure S4: MIAT regulates RGC function upon hypoxia stress. (**A**) RGCs were transfected with scrambled siRNA (Scr), MIAT siRNA, or left untreated, and then exposed to hypoxia ($CoCl_2$, 200 μm) for 48 h. Cell viability was detected using MTT method (n = 4). (**B**) Apoptotic cells were analyzed using Hoechst staining and quantitated (n = 4). (**C**) RGCs were incubated with JC-1 probe at 37°C for 30 min, centrifuged, washed, transferred to a 96-well plate (100, 000 cells per well), and assayed using a fluorescence plate reader (n = 4).

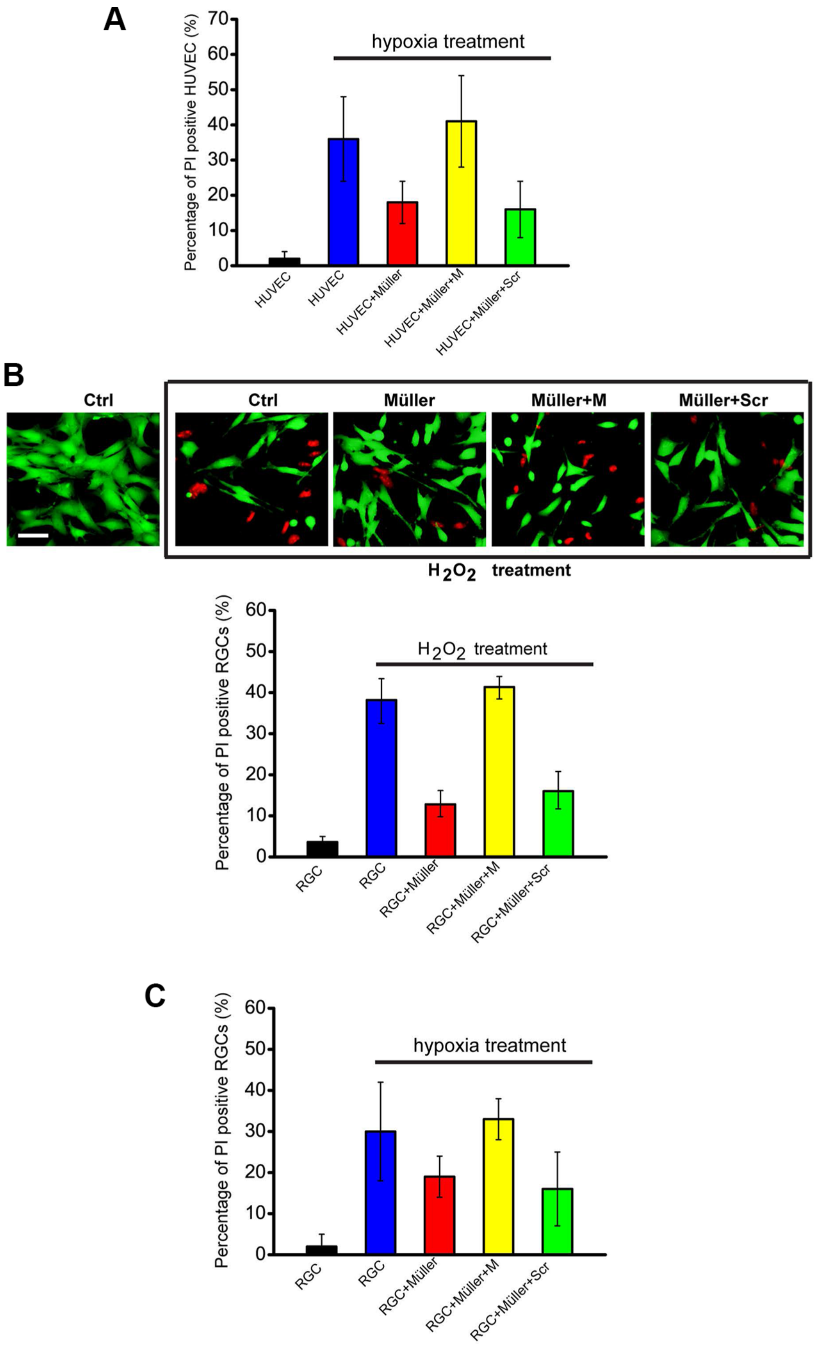

Supplementary Figure S5: MIAT knockdown in Müller cells affects HUVEC and RGC function. (**A**) HUVECs were co-cultured with Müller cells, and then treated with hypoxia ($CoCl_2$, 200 μm) for 48 h. PI staining and quantitative analysis was conducted to detect the dead or dying cells (*n* = 4). (**B**, **C**) RGCs were co-cultured with Müller cells, and then treated with H_2O_2 (50 μm, B) or hypoxia ($CoCl_2$, 200 μm, C) for 48 h. PI staining and quantitative analysis was conducted to detect the dead or dying cells (*n* = 4). Scale bar, 20 μm.

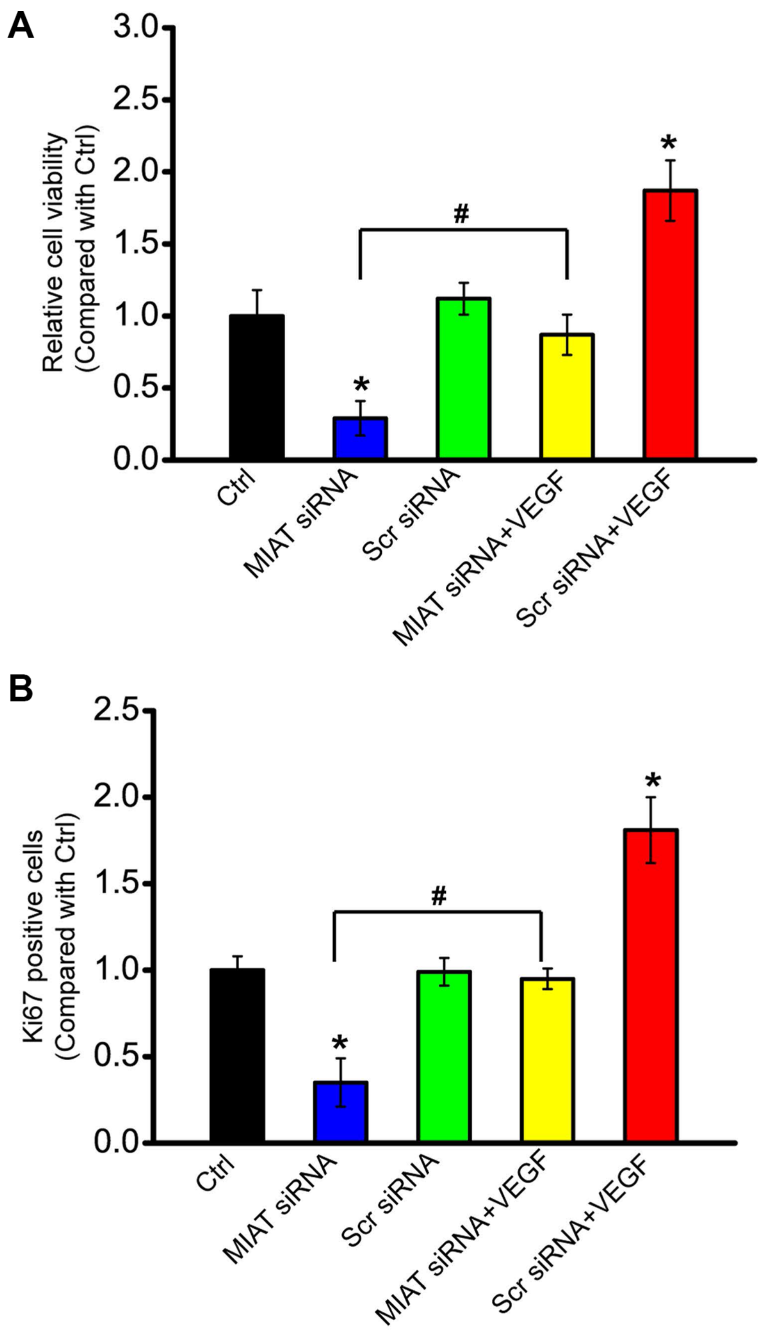

Supplementary Figure S6: MIAT-miR-150-5p regulatory loop is critical for Müller cell function. (**A**) MTT assay was performed to detect Müller cell viability. The data was expressed as relative change compared with the control group (n =4). (**B**) A representative image was shown for Ki67 staining of Müller cells along with the quantification of Ki67 positive cells upon different treatments. Scale bar, 20 μm. "*" indicated significant difference compared with the corresponding control group. "#" indicated significant difference between different experimental groups.

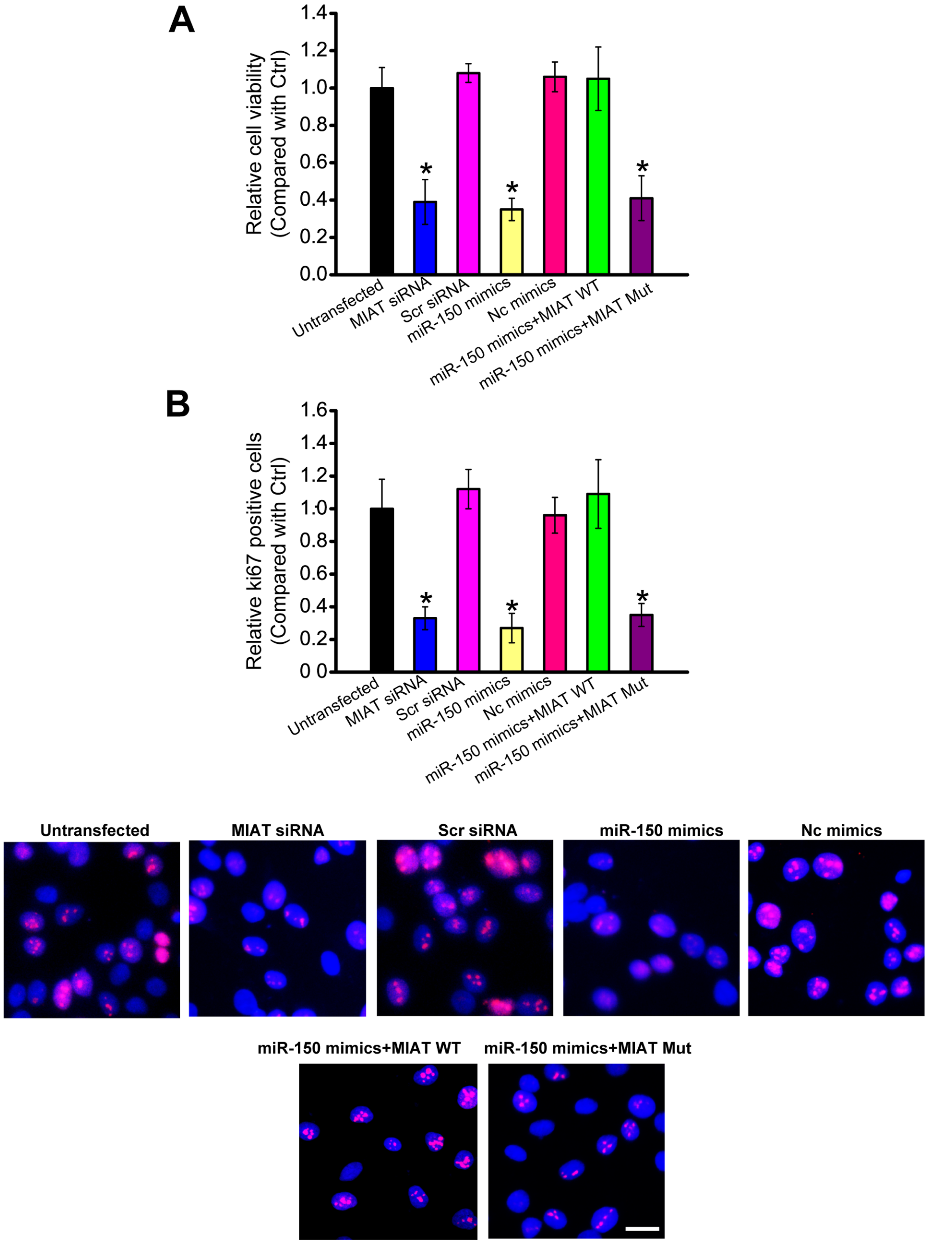

Supplementary Figure S7: MIAT-VEGF crosstalk is involved in regulating Müller cell function. (**A**, **B**) Müller cells were transfected with MIAT siRNA or scrambled siRNA, and then treated with or without VEGF (10 ng/ml) for 24 h. Cell viability was detected using MTT assay (**A**). Cell proliferation was detected using ki67 staining, and then quantified (**B**), $n = 4$, *$P < 0.05$). "*" indicated significant difference compared with the corresponding control group. "#" indicated significant difference between the marked groups.

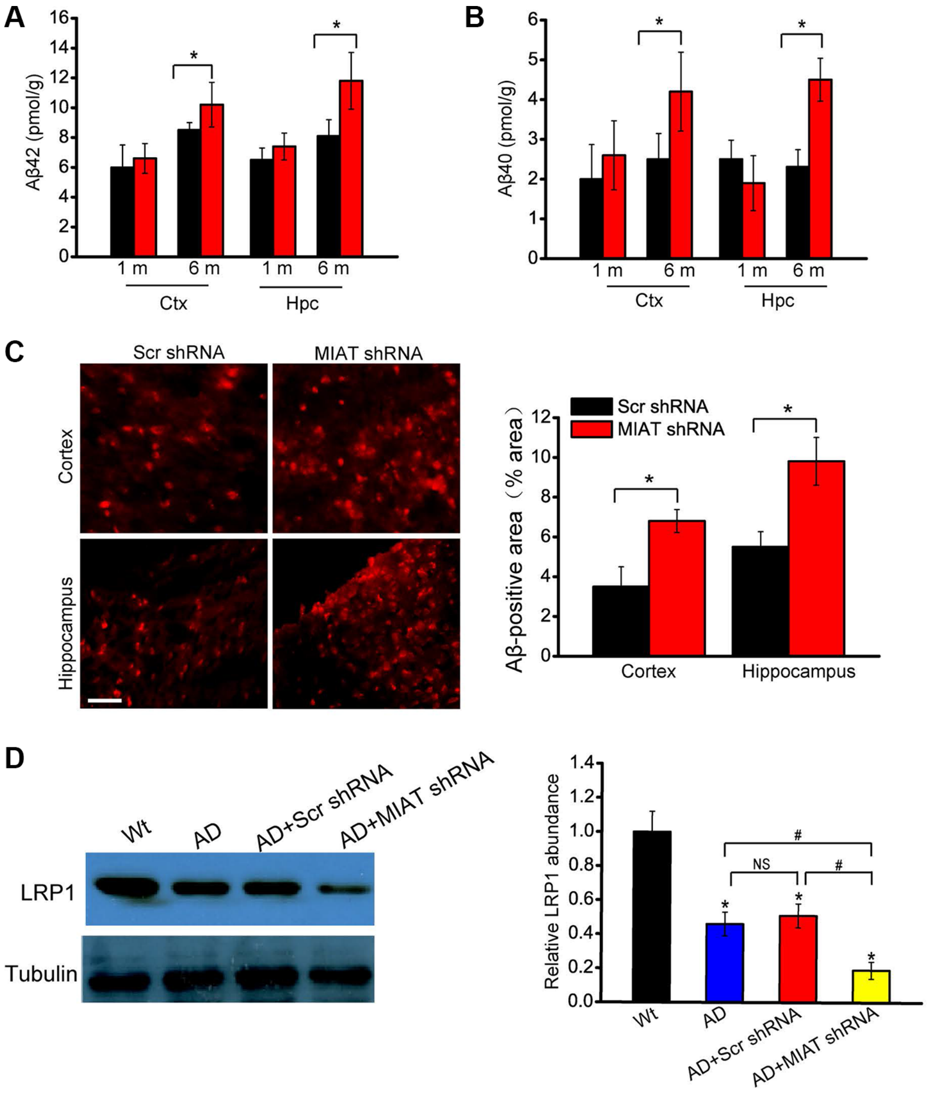

Supplementary Figure S8: MIAT knockdown affects the progress of CNS β-amyloidosis. (**A**, **B**) Cortical (Ctx) and hippocampal (Hpc) Aβ40 (A) and Aβ42 (B) levels were detected in 1-, and 12-month-old APP/PSN mice. $n = 6$ mice per group; *$P < 0.05$. (**C**) Aβ-immunodetection (red) and quantification in the somatosensory cortex and CA1 hippocampal subfield in MIAT knockdown mice and age-matched littermates. Scale bar, 200 μm. $n = 6$ mice per group; *$P < 0.05$. (**D**) Immunoblotting for LRP1 in isolated microvessels (top panel) and quantification (bottom panel) in 12-month-old MIAT knockdown mice and age-matched littermates. $n = 6$ mice per group; *$P < 0.05$, NS, no significant difference.
